# Imitators of exercise-induced bronchoconstriction

**DOI:** 10.1186/1710-1492-5-7

**Published:** 2009-11-17

**Authors:** Pnina Weiss, Kenneth W Rundell

**Affiliations:** 1 Department of Pediatrics, Yale School of Medicine, P.O. Box 208064, New Haven, CT, 06520-8064, USA; 2 Center for Healthy Families, Respiratory Research, Marywood University, 2300 Adams Avenue, Scranton, PA, 18509-1598, USA

## Abstract

Exercise-induced bronchoconstriction (EIB) is described by transient narrowing of the airways after exercise. It occurs in approximately 10% of the general population, while athletes may show a higher prevalence, especially in cold weather and ice rink athletes. Diagnosis of EIB is often made on the basis of self-reported symptoms without objective lung function tests, however, the presence of EIB can not be accurately determined on the basis of symptoms and may be under-, over-, or misdiagnosed. The goal of this review is to describe other clinical entities that mimic asthma or EIB symptoms and can be confused with EIB.

## Diagnosis of exercise-induced bronchoconstriction

Exercise-induced bronchoconstriction (EIB) is a common entity and is described by the transient narrowing of the airways during or most often after exercise [1-4]. It occurs in 10-15% of the general population [5,6], while the prevalence of EIB in asthmatic patients is reported to be 80-90% [7-9]. Athletes generally show a high prevalence of EIB [10,11], especially in the cold weather [12-15], and ice rink athletes demonstrate a much greater prevalence of EIB than their non-ice rink counterparts [16-19]. In varsity college or elite athletes, 21-50% demonstrate EIB, depending upon the specific sport demands [11,20-23]

The diagnosis of EIB is often made on the basis of self-reported symptoms without objective lung function tests. However, the presence of EIB can not be accurately determined on the basis of symptoms [20,23,24]. Recent studies demonstrate a lack of sensitivity and specificity of the symptoms-based diagnosis [23]. In one study of elite athletes, 39% of athletes positive to exercise challenge reported two or more symptoms, while 41% of those negative reported 2 or more symptoms [24]. In fact, history is little more reliable than flipping a coin in making the diagnosis of EIB.

Accurate diagnosis of EIB is essential. EIB is effectively prevented by acute use of b2-agonists, leukotriene receptor antagonists, sodium cromoglycate, and nedocromil sodium and the chronic use of inhaled corticosteroids [7,25,26]. However, these medications are often needlessly prescribed for patients who do not have EIB; they are often used in combination when such patients do not respond to first-line therapy. Because of the high b2-agonist use among the elite athletes, the International Olympic Committee (IOC) requires objective evidence to demonstrate asthma or EIB as an indication for therapeutic use of b2-agonists during competition [27,28].

**Figure 1:**
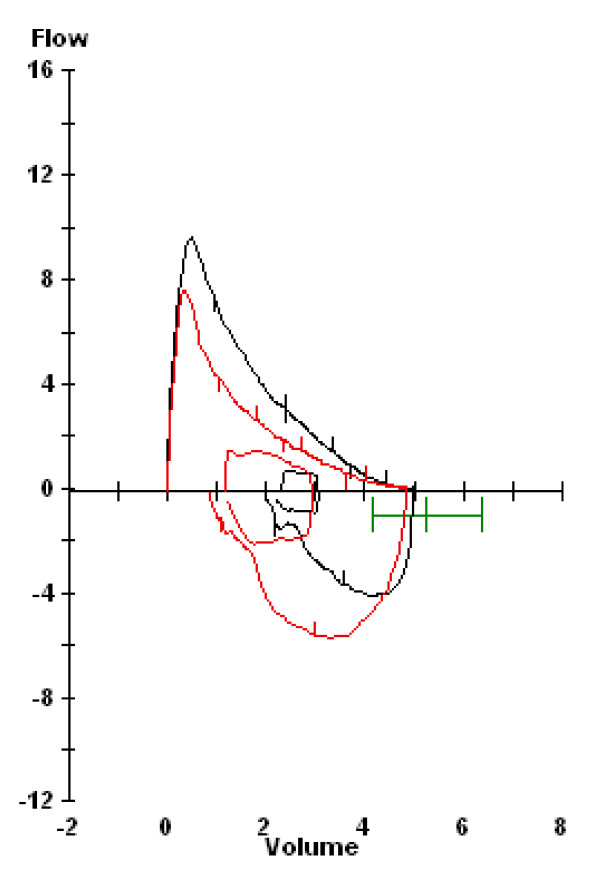
**Pre- and post-exercise spirogram demonstrating a 19% fall in FEV1**. A ≥ 10% fall is indicative of EIB.

Therefore, confirmation of the diagnosis of EIB through standardized testing utilizing spirometry should be performed. Current guidelines (ATS, ERS, IOC-MC) for a diagnosis of EIB require a 10% or greater decrease in forced expiratory flow in the first second of exhalation (FEV_1_) in response to exercise (Figure 1) or eucapnic voluntary hyperpnea (EVH).

Symptoms of EIB include dyspnea (sensation of discomfort when breathing), increased effort or work to breathe, chest tightness, shortness of breath, air hunger, wheezing, or cough [24,29]. However, other clinical entities can produce similar symptoms [30]. Dyspnea, in particular, is associated with many disease processes [31,32]. In fact, EIB is uncommon in subjects who complain of exercise-induced dyspnea. In patients who presented with exercise induced dyspnea, only 7-24% actually had EIB on cardiopulmonary testing [6,33]. "Wheeze" or stridor can also be caused by airway abnormalities and may closely mimic EIB.

The goal of this review is to describe other clinical entities that mimic asthma or EIB symptoms and can be confused with it. More than one condition may coexist in a given patient.

## Physiologic limitation and deconditioning

Increased ventilation is a normal physiologic response to exercise. However, the increase in respiratory drive and work may be interpreted as pathologic by subjects who find that it limits their ability to perform to their expectations or results in "normal" discomfort. In a study by Abu-hasan et al, physiologic limitation was the most common reason for exercise-induced dyspnea in pediatric patients who underwent cardiopulmonary exercise testing [34]. It occurred in 52% of referrals for EIB; of those, two thirds had normal or above normal cardiovascular conditioning. The dyspnea is likely related to the increase in ventilation that accompanies high intensity exercise which is necessary to meet increased metabolic demands. Minute ventilation and respiratory drive are further increased at or above the lactate or ventilatory threshold, the point in incremental exercise when lactate begins to accumulate in the serum; excess lactate buildup results in exercise-associated increases in ventilation and ultimately hypocapnia. Subjects perceive dyspnea and shortness of breath at these high exercise intensities as abnormal.

Deconditioned subjects have a lower lactate/ventilatory threshold and begin to accumulate lactate and increase minute ventilation with lesser amounts of exercise. Deconditioning is a common etiology for exercise-induced dyspnea [32]. In a study by Seear et al., 23% of patients were "unfit" [6]. In Abu-Hasan's study, roughly 17% had decreased cardiovascular conditioning [34]. An athlete who has become deconditioned during the "off season" may interpret an increase in respiratory drive with lesser amounts of exercise as pathologic or as "EIB" or asthma.

Exercise rehabilitation or training can improve aerobic fitness and endurance [8] and can shift the lactate/ventilatory threshold so more work is required before lactate accumulates and ventilation increases. Improved aerobic fitness through exercise training can thus decrease the hyperpnea and dyspnea associated with exercise [35-37].

## Obesity

Exercise-induced dyspnea is very common in obese patients. In one epidemiological study, 80% of obese middle aged subjects reported dyspnea after climbing two flights of stairs [38]. In another study, 36.5% of obese adults with a body mass index (BMI) greater than 31 and 28% of "overweight" adults (BMI 27-31) reported dyspnea when walking up hill [39]. In formal cardiopulmonary exercise testing, 37% of healthy obese women had an elevated perception of breathlessness during exercise [40].

There are several reasons why obese subjects experience dyspnea during exercise. Obesity is associated with impairment of pulmonary mechanics; in mild obesity, there is a reduced expiratory reserve volume (ERV) which is likely due to the displacement of the diaphragm into the chest cavity by the fat stores within the abdomen. With increasing severity of obesity, there are decreases in total lung capacity (TLC), functional residual capacity (FRC), and maximal voluntary ventilation (MVV)[41-44]. Because of the lower lung volumes, there may be a decrease in airway caliber and increase in airway resistance. The chest wall and total respiratory system are less compliant, which increases the work and energy cost of breathing [45]. The decrease in end expiratory lung volume likely causes flow limitation during exercise.

Aerobic capacity and cardiopulmonary fitness may be decreased in obese patients. Cardiopulmonary fitness, reflected by maximal or peak oxygen consumption (VO_2 _max) is decreased when corrected for weight [46]. Obese subjects are very likely to be deconditioned and ventilatory threshold may be reduced. Cardiac performance in response to incremental work load may also be decreased [47].

A number of studies have demonstrated that obese subjects perceive a greater degree of dyspnea in response to stimuli such as exercise, methacholine challenges or asthma exacerbations [39,48,49]. In one study in obese women, the degree of exercise-induced dyspnea was directly correlated to increases in the oxygen cost of breathing [40]. Weight loss can improve the pulmonary mechanics and lung volumes in these patients [43,50].

## Vocal cord abnormalities

Obstruction of the upper airway can cause symptoms such as shortness of breath, increased inspiratory effort, stridor and wheeze. In many subjects, upper airway obstruction is dynamic and only presents during exercise.

Paradoxical vocal cord movement is the most common cause of upper airway obstruction during exercise [51]. Typically, during inspiration, the vocal cords abduct (open); however, in some subjects, they paradoxically adduct (close) during inspiration or early expiration which causes obstruction. The prevalence of vocal cord dysfunction (VCD) has been reported to range from 5 -15% in patients referred for exercise-induced dyspnea [34,52,53]. However, in one study, the incidence was as high as 27% [6].

Diagnosis may be suspected by history of inspiratory wheeze and throat tightness. VCD has been associated with gastroesophageal reflux [54] and "type A personalities" [Weiss, unpublished observation]. Prevalence of VCD appears to be gender related and is highest among young females [53]. In a study of 370 (174 female, 196 male) elite athletes by Rundell and Spiering, 30% (58 female, 53 male) tested positive for EIB and 5.1% demonstrated inspiratory stridor consistent with VCD; of those, 18 of 19 were females [53]. Ten of those demonstrating inspiratory stridor were positive for EIB. Eight of the 9 demonstrating stridor that were negative for EIB had a previous diagnosis of EIB and 7 of those were prescribed albuterol by their physician, with no resolution of stridor.

The diagnosis of VCD is suggested by flow-volume loops which may reveal variable blunting of the inspiratory loop. In one study, 60% of VCD-positive patients developed abnormal flow-volume loops after metacholine challenge [52]. Definitive diagnosis can be made by fiberoptic rhinolaryngoscopy, which reveals the paradoxical motion of the vocal cords. The typical findings from laryngoscopy are inspiratory vocal cord closure with posterior "chinking" (a small opening at the posterior aspect of the cords) or, less commonly, complete closure [55,56].

VCD may respond to breathing retraining diaphragmatic breathing - relaxation of larynx with conscious activation of the diaphragm [57,58]. Speech pathologists are often an invaluable resource in providing subjects with instruction on breathing training exercises.

Laryngomalacia is less common cause of exercise-induced stridor. It primarily affects female competitive athletes who abruptly develop stridor at near peak exercise [59]. It is differentiated from vocal cord dysfunction by fiberoptic rhinolaryngoscopy. It is characterized by collapse of the arytenoid area; vocal cord motion is normal. The larynx in females may be predisposed to collapse, because it is shorter and narrower than in males. One reported patient had a history of laryngomalacia as an infant [60]. Laryngomalacia has been successfully treated with laser supraglottoplasty [61,62].

## Anxiety and Hyperventilation Syndrome

Anxiety may produce a heightened sense of breathlessness and dyspnea during exercise. Hyperventilation is a common physiologic response to both exercise and anxiety but may be interpreted as a primary problem that could be associated with chest tightness and shortness of breath [63]. In severe cases, it may be associated with carpopedal spasm, tetany and seizures [64]. In fact, in the past it was suggested that patients with panic and anxiety disorders actually had inherent respiratory and autonomic abnormalities. More recently, the entity of primary hyperventilation syndrome has been deemed a "chimera" [65]; that it is "no longer tenable." It is more likely that hyperventilation is a result of the panic attacks and associated anxiety [65-67].

The emotional state of subjects may impact their perception of dyspnea. In subjects with high levels of anxiety and multiple somatic complaints, there is an exaggerated perception of the intensity of dyspnea when hyperventilation is evoked by breathing 5% CO2 enriched air [68,69]. In asthmatic patients stress, negative emotions and fear or anticipation increase subjective reports of dyspnea [70-72].

In subjects with a high level of anxiety, who have an exaggerated sense of dyspnea during exercise, it would be worthwhile to perform cardiopulmonary exercise testing and document the absence of EIB. In many cases, reassurance that the response to exercise is normal may allay anxiety and improve symptoms. The power of positive suggestion plays an important role in the relief of dyspnea and pain perception by decreasing anxiety [73]. Breathing retraining exercises may be helpful to decrease hyperventilation and self-hypnosis has been effective in reducing dyspnea in pediatric subjects [74]. In severe cases, pharmacologic therapy for anxiety may be indicated.

## Cardiac abnormalities

In previously healthy persons, cardiac abnormalities are a rare cause of exercise-induced dyspnea. In older patients with cardiovascular diseases, particularly congestive heart failure, exercise performance is limited because of decreases in cardiac and pulmonary reserve. Patients may hyperventilate and experience dyspnea at lower work loads because of earlier onset of metabolic acidosis, decreased lung compliance and increased airways resistance because of pulmonary edema and increased dead space ventilation [75]. The ventilatory response to exercise can be improved by treatment of the underlying heart failure [76].

Pulmonary vascular diseases, such as pulmonary hypertension, can be associated with dyspnea, cardiac limitation and abnormal ventilatory responses to exercise [77]. Pulmonary hypertension may be associated with lower airways obstruction and increased airways hyperreactivity [78,79]. In rare cases, it may present as refractory asthma because of extrinsic proximal airway obstruction by dilated pulmonary arteries [80]. Diagnosis is usually made on the basis of cardiac echocardiography and catheterization.

Hypertrophic cardiomyopathy (HCM), a feared cause of sudden death in athletes, can be associated with exercise-induced dyspnea and progressive heart failure [81-84]. It would be unlikely, but not impossible for it to present as "exercise-induced asthma." Those at highest risk of sudden death are subjects with a history of cardiac arrest or ventricular tachycardia, family history of HCM-related death, syncope, or left ventricular hypertrophy [82]. All athletes who are screened by a pre-sports participation physical should be asked about risk factors.

Cardiac dysrythmias are a rare cause of exercise-induced dyspnea. Atrial fibrillation and other supraventricular tachyarrhythmias are uncommon in elite athletes and similar to that observed in the general population (< 1%) [85]. Tachyarrythmias are often associated with palpitations or, rarely, syncope. Abu-Hassan et al reported one teenager who developed supraventricular tachycardia as a cause of his exercise-induced dyspnea; of note, he did not complain of palpitations [34]. Atrioventricular block could potentially cause exertional dyspnea [86-88]. Patients may present with exercise intolerance and AV block from Lyme disease [89]; we have documented one pediatric patient with exercise intolerance attributed to a complete AV block from Lyme disease [Weiss, unpublished observation].

Vascular anomalies of the thoracic aorta such as a double aortic arch or right aortic arch with persistent ligamentum arteriosum or aberrant left subclavian artery have been associated with dyspnea on exertion [90,91]. The mechanisms for the symptoms include associated tracheomalacia and extrinsic compression of the airways which may worsen during exercise because of aortic arch dilatation. Surgical correction may be necessary.

## Pulmonary arteriovenous malformations

Pulmonary arteriovenous malformations (AVM) can be associated with exercise-intolerance and arterial hypoxemia. Most pulmonary AVMs are associated with an autosomal dominant disorder, hereditary hemorrhagic telangiectasia (HHT) or Osler-Weber Rendu [92]. The incidence of HHT is estimated to be greater than one in 10,000 [93]; approximately 35% of patients with HHT have pulmonary AVMs [94]. The complications of pulmonary AVMs are related to the intrapulmonary right-to-left shunt. Paradoxical emboli can result in cerebral abscesses, cerebrovascular accidents and transient ischemic attacks [93,95]. Most pulmonary AVMs are located at the lung bases. Some patients demonstrate platypnea or improvement in breathing on reclining [96]. Arterial hypoxemia which is worse in the upright position or with exercise is common [97]. Spirometry is usually normal, however, diffusing lung capacity for carbon monoxide (DLCO) may be decreased [97-100].

The gold-standards for diagnosis of AVMs are pulmonary angiography and chest computed tomography [101,102]. Chest radiography, arterial oxygen measurements, cardiopulmonary exercise testing, radionuclide lung scanning, contrast-enhanced MR angiography and transthoracic contrast echocardiography (TTCE) have been used as screening methods [103-108]. Transcatheter embolization is the therapy of choice and has been shown to decrease the right to left-shunt and improve arterial hypoxemia and exercise tolerance [97-100].

## Pulmonary abnormalities

Other pulmonary abnormalities can present with exercise-induced dyspnea. Chest wall or other musculoskeletal abnormalities can impair pulmonary mechanics. In the series of Abu-Hasan et al, 11% of patients had restrictive physiology due to mild scoliosis or pectus abnormality as the cause of their exercise-induced dyspnea [34]. Pectus excavatum has been associated with exercise intolerance and dyspnea; improvement after surgical correction has been documented [109-111]. Mild scoliosis in adolescents has been associated with abnormal ventilatory response to exercise [112]. In contrast, in adults with moderate kyphoscoliosis, dyspnea has been attributed to deconditioning rather than disordered pulmonary mechanics [113].

Tracheobronchomalacia, dynamic collapse of the central airways, may produce airflow limitation during exercise and has been associated with exercise intolerance [114]. The incidence of malacia has been estimated to be in 1: 2,100 children [115]. The symptoms overlap with those of asthma and it is often unsuspected until documented by bronchoscopy.

Interstitial lung disease is associated with exercise-induced dyspnea. Mechanisms for dyspnea and exercise limitation are expiratory flow limitation, hypoxemia and altered pulmonary mechanics [116-118]. Diagnosis may be made on the basis of pulmonary function tests revealing restrictive physiology, decreases in diffusing lung capacity for carbon monoxide (DLCO), chest CT, serum serology, bronchoscopy and/or lung biopsy.

The sequelae of moderate and severe chronic obstructive pulmonary disease (COPD) on exercise-induced dyspnea are well recognized. However, mild COPD can also be associated with increased dyspnea with exertion which reflects abnormal ventilatory mechanics including airway obstruction, increases in end-expiratory lung volumes and deconditioning [119-122].

## Myopathy

Dyspnea can be associated with diseases of skeletal muscles (myopathies) [123]. In muscular dystrophies, there is a progressive loss of muscle fibers which results in increasing muscle weakness. In disorders of muscle energy metabolism, there is an imbalance in muscle energy production and utilization during exercise which can result in exertional muscle pain, cramping, weakness, or fatigue. Mitochondrial myopathy is an often unrecognized cause of exertional dyspnea and exercise intolerance [124,125]. Flaherty et al, described 28 patients with biopsy proven myopathy and found that exercise dyspnea was associated with decreased respiratory muscle function [126]. Cardiopulmonary exercise testing revealed mechanical ventilatory limitation and an exaggerated increase in respiratory frequency and tachycardia in response to exercise. Many patients had respiratory muscle weakness.

## Summary

**Figure 2:**
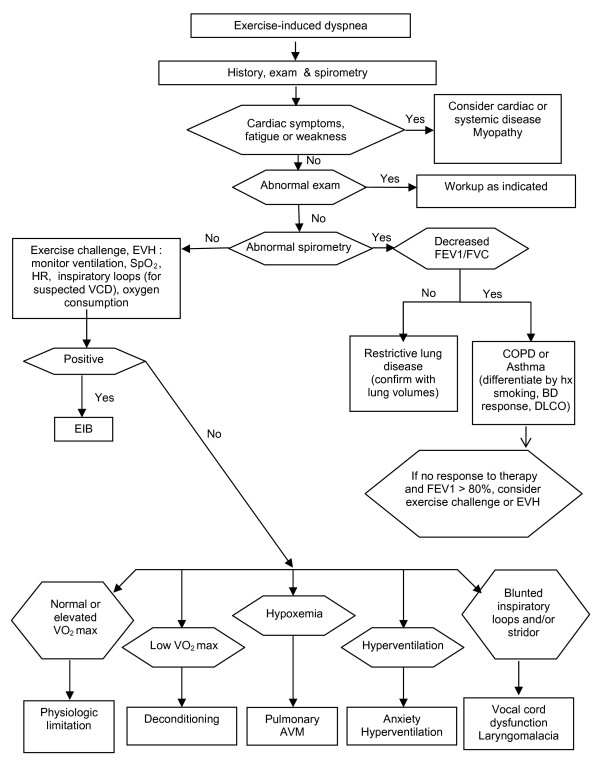
**Differential diagnosis algorithm for exercise-induced dyspnea**.

In summary, reported symptoms and history without objective lung function tests are not adequate to make a definitive diagnosis of EIB. Approximately half of those athletes reporting symptoms of EIB have normal airway function and about half of those who report no symptoms will demonstrate bronchoconstriction after exercise or other indirect challenge [20,23,24,127]. It is therefore important to confirm a diagnosis of EIB through objective measures of lung function using standardized procedures. Indirect challenges such as exercise, eucapnic voluntary hyperpnea (EVH) or inhaled powdered mannitol are more specific to EIB than direct challenges such as histamine or methacholine [128,129]. Figure 2 provides an algorithm for differential diagnosis of EIB. Differential diagnosis of EIB should include normal physiologic limitation and deconditioning, obesity, upper airway obstruction such as vocal cord dysfunction or laryngomalacia, anxiety-associated dyspnea and hyperventilation, exercise-induced supraventricular tachycardia, as well as other cardiac and pulmonary abnormalities.

## Competing interests

The authors declare that they have no competing interests.

## Authors' contributions

Both authors have made substantive contributions to drafting and revising the manuscript. Both authors read and approved the final manuscript.
